# Endovascular management of dissecting posterior cerebral artery aneurysm associated with persistent hypoglossal artery: A case report

**DOI:** 10.1590/1677-5449.200142

**Published:** 2021-08-02

**Authors:** Vivek Murumkar, Sameer Peer, Jitender Saini, Hanumanthapura Ramalingaiah Arvinda

**Affiliations:** 1 Department of Neuroimaging and Interventional Radiology, National Institute of Mental Health and Neurosciences (NIMHANS), Bengaluru, Karnataka, India.

**Keywords:** persistent hypoglossal artery, carotid-basilar anastomosis, dissecting aneurysm, artéria hipoglossa persistente, anastomose carotídeo-basilar, aneurisma dissecante

## Abstract

Persistent embryological connections between the anterior and posterior circulations are rare entities. Persistent hypoglossal artery is the second most common persistent carotid-basilar anastomosis. As it is often associated with hypoplasia of vertebral arteries, it poses a challenge during endovascular interventions. We present a case of a 32-year-old woman who presented with occipital headache of four weeks’ duration. Magnetic Resonance Angiography showed hypoplastic vertebral arteries with a persistent hypoglossal artery arising from the cervical segment of the left internal carotid artery and supplying the entire posterior circulation, associated with a dissecting aneurysm of the right posterior cerebral artery. Endovascular parent vessel occlusion was performed for the dissecting posterior cerebral artery aneurysm by navigating the guide catheter, microwire, and microcatheter through the persistent hypoglossal artery because the vertebral arteries were hypoplastic. Post-intervention, the patient did not develop any neurological deficit and was discharged in a stable condition.

## Resumo

Conexões embriológicas persistentes entre as circulações anterior e posterior são entidades raras. A artéria hipoglossa persistente é a segunda anastomose carotídeo-basilar persistente mais comum. Como está frequentemente associada à hipoplasia das artérias vertebrais, apresenta um desafio durante as intervenções endovasculares. Apresentamos o caso de uma mulher de 32 anos que apresentou cefaleia occipital com duração de quatro semanas. A angiografia por ressonância magnética mostrou artérias vertebrais hipoplásicas com artéria hipoglossa persistente surgindo do segmento cervical da artéria carótida interna esquerda e suprindo toda a circulação posterior com um aneurisma dissecante da artéria cerebral posterior direita. A oclusão endovascular do vaso parental foi realizada para o aneurisma da dissecção da artéria cerebral posterior pela passagem de cateter guia, microfio e microcateter pela artéria hipoglossa persistente, pois as artérias vertebrais eram hipoplásicas. Após a intervenção, a paciente não apresentou déficit neurológico e recebeu alta em uma condição estável.

## INTRODUCTION

During the embryonic stage, the forebrain is supplied by a pair of primitive internal carotid arteries and the hindbrain is supplied by the paired longitudinal neural arteries. There are four anastomotic channels connecting the primitive internal carotid artery and longitudinal neural arteries, namely, the trigeminal artery, the otic artery, the hypoglossal artery, and the proatlantal intersegmental artery.^1^ With the development of the posterior communicating arteries and the vertebral arteries, these anastomotic channels involute.^1^^,^^2^ Failure of regression of these channels will lead to formation of persistent carotid-basilar anastomosis, the most common of which is the persistent trigeminal artery.^3^ Persistent hypoglossal artery (PHA) is the second most common persistent carotid-basilar anastomosis with a reported incidence of 0.1-.02%.^1^ PHA is associated with intracranial aneurysms, atherosclerotic changes, and subsequent posterior circulation ischemia.^3^^-^5 We hereby present a case of dissecting posterior cerebral artery aneurysm associated with PHA. Endovascular coiling with parent vessel occlusion (PVO) was performed for the aneurysm. Post-coiling, the patient did not develop any neurological deficits and she was discharged in a stable condition. This manuscript is in accordance with the Helsinki declaration. Informed consent for the publication of this manuscript was obtained in writing from the patient.

## CASE DESCRIPTION

**Figure 1:**
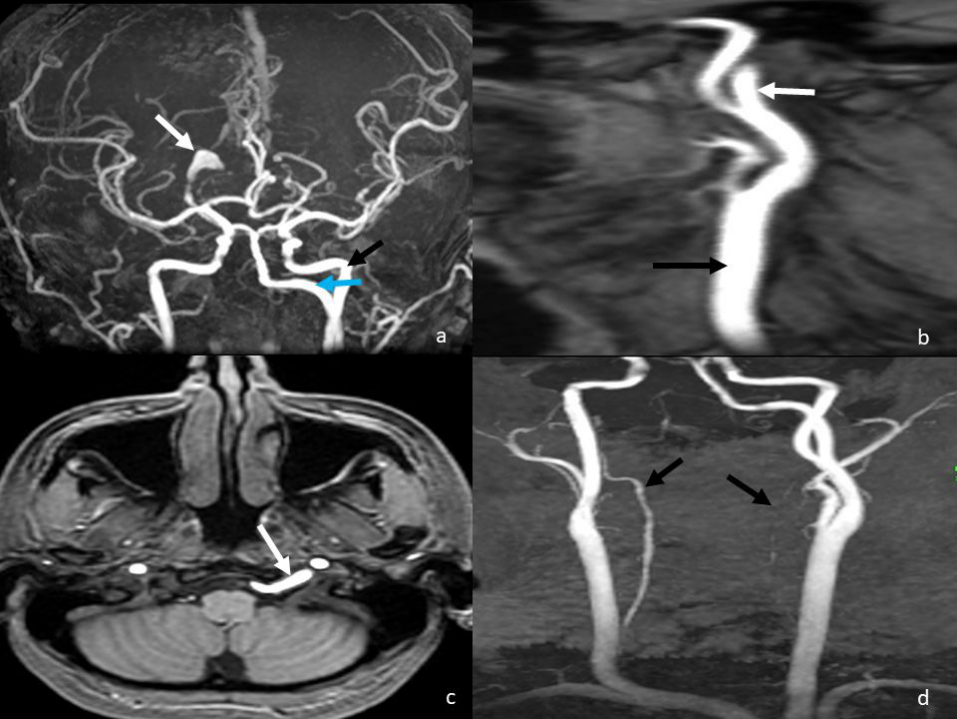
Diagnosis of the dissecting aneurysm of the right posterior cerebral artery associated with persistent hypoglossal artery. **a)** Maximum intensity projection magnetic resonance angiogram image showing a dissecting aneurysm involving the P2-P3 segment of the right posterior cerebral artery (white arrow). A persistent hypoglossal artery (blue arrow) is noted medial to the left internal carotid artery (black arrow) and joining the basilar trunk. **b)** Lateral maximum intensity projection magnetic resonance angiogram showing the persistent hypoglossal artery (white arrow) arising from the dorsal aspect of the left internal carotid artery (black arrow). **c)** Axial magnetic resonance angiogram section at the level of the hypoglossal canal showing the persistent hypoglossal artery (white arrow) traversing the left hypoglossal canal. **d)** Maximum intensity projection magnetic resonance angiogram of the neck showing bilateral hypoplasia of vertebral arteries (black arrows).

A 32-year-old female presented with mild occipital headache of four weeks’ duration. She did not have any focal deficits. Neurological examination was normal. Magnetic Resonance Imaging (MRI) showed evidence of an aneurysm, 2.1 x 1.5 cm in size, involving the right posterior cerebral artery at the P2-P3 junction (Figure 1A). There was no evidence of hemosiderin staining to suggest subarachnoid hemorrhage. An anomalous vascular channel was found to arise from the left cervical internal carotid artery at the C1-C2 junction (Figure 1B), coursing through the left hypoglossal canal along with the hypoglossal nerve (Figure 1C). It was seen joining the basilar trunk and reforming it. Vertebral arteries were found to be hypoplastic bilaterally (Figure 1D). Subsequently, a diagnosis of PHA was made. It was decided to treat the aneurysm with endovascular parent vessel occlusion. Since both the vertebral arteries were hypoplastic it was decided to navigate through the PHA.

**Figure 2:**
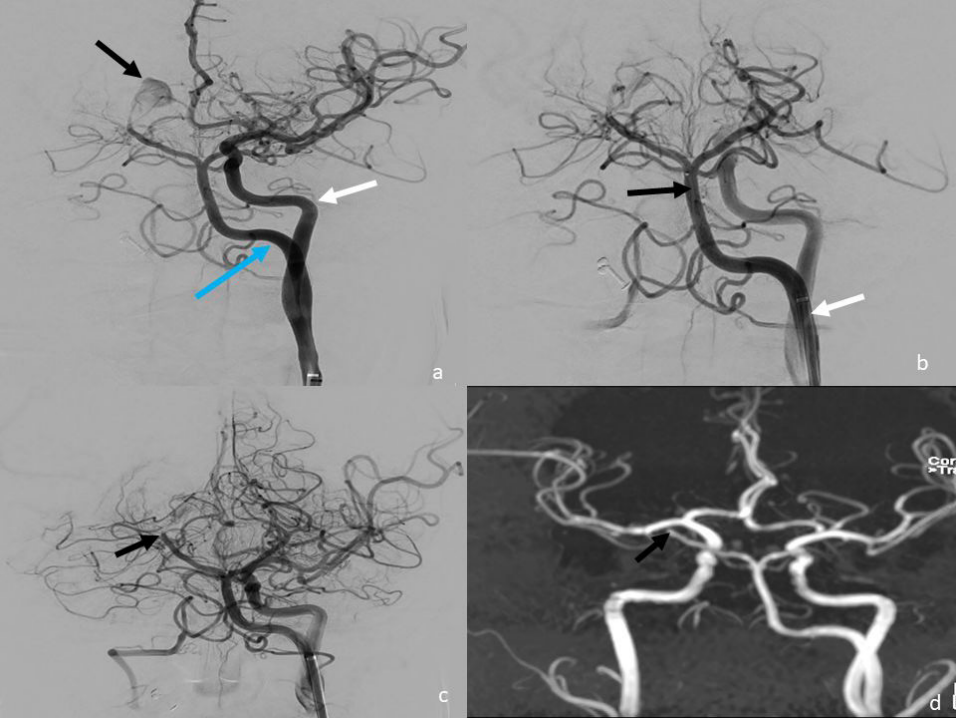
Endovascular approach through the persistent hypoglossal artery for occlusion of the right P2-P3 segment. **a)** Digital subtraction angiogram with the guide catheter in the left internal carotid artery (white arrow) showing the persistent hypoglossal artery (blue arrow) arising from the cervical segment of the left internal carotid artery and supplying the posterior circulation. A dissecting aneurysm (black arrow) is noted involving the P2-P3 segment of the right posterior cerebral artery with narrowing of the segment proximal to the aneurysm. **b)** Digital subtraction angiogram with guide catheter in the persistent hypoglossal artery (white arrow). The microcatheter (black arrow) was navigated through the PHA into the right posterior cerebral artery to reach the desired location for parent artery occlusion. **c)** Parent artery occlusion of the P2-P3 segment of the right posterior cerebral artery with coils (black arrow). No residual filling of the aneurysm is seen. **d)** Maximum intensity projection of the magnetic resonance angiogram showing no residual filling of the aneurysm with occlusion of the P2-P3 segment of the right posterior cerebral artery (black arrow).

The procedure was performed under general anesthesia. The left femoral artery was punctured with an 18G needle and the access was secured with an 8F short sheath. 5000 IU of heparin was given intravenously as a bolus after which 1000 IU was repeated hourly till the end of the procedure. The Neuron Max long sheath (Penumbra Inc. USA) 5F diagnostic catheter-0.035 guide wire coaxial system was taken into the PHA under road-map guidance. The Neuron max was connected to continuous nimodipine flush. Angiogram was obtained, showing an aneurysm involving the right P2-P3 posterior cerebral artery with proximal narrowed segment (pearl and string sign) suggestive of dissecting aneurysm (Figure 2A). It was decided to perform PVO. A working projection was established. An excelsior SL-10 straight tip microcatheter (Stryker Neurovascular, USA) was taken over synchro 0.014” microwire (Stryker Neurovascular, USA) and the right posterior cerebral artery was cannulated (Figure 2B). The catheter was navigated into the P2-P3 junction, just proximal to the aneurysm and the position was confirmed fluoroscopically. Two coils of size 2.5 mm x 6 cm and 2 mm x 4 cm (Axium prime Medtronic, CA) were deployed sequentially into the parent vessel (P2/P3 segment of right posterior cerebral artery). Check angiogram showed significant stasis of blood flow in the aneurysm. There was normal filling of other arteries of the posterior circulation (Figure 2C and 2D).

Immediate post-procedure Computed Tomography (CT) did not show any fresh bleeding or infarct. The patient had symptomatic relief of headache over the next few days. She did not develop any new focal neurological deficits after the procedure and discharged in a stable condition. At follow-up after 2 months, the patient did not complain of headache. Her neurological examination at follow-up was normal.

## DISCUSSION

Carotid-basilar anastomosis exists in early embryonic life (4-5 mm stage), connecting the primitive ICA and LNA.^1^ There are four transient anastomotic channels, namely the trigeminal artery, otic artery, hypoglossal artery, and proatlantal artery (in craniocaudal order). Padget published a detailed evaluation of these anastomotic channels in 1948.^1^ Subsequently, development of a posterior communicating artery and fusion of cervical intersegmental arteries gives rise to the vertebral arteries, leading to regression of these anastomotic channels. The otic artery is first to regress and the last to regress is the trigeminal artery, which is the most common persistent carotid-basilar anastomosis.^6^ The second most common carotid-basilar anastomosis is the PHA with an incidence of about 0.1-0.2% on cerebral angiography.^7^ It usually arises from the cervical segment of the internal carotid artery at the C1-2 level, traverses through the hypoglossal canal and joins the basilar artery, and is often the only supply to the posterior circulation because one or both of the vertebral arteries are hypoplastic.^1^^,^^6^^,^^8^

PHA can be asymptomatic and may be diagnosed as an incidental finding. It is critical to identify this artery before carotid endarterectomy and skull base surgeries as this may be the only vessel supplying the entire posterior circulation. PHA is also found to be associated with intracranial aneurysms, atherosclerosis, and posterior circulation ischemia.^5^^,^^7^^,^^9^ Atherosclerotic changes may occur as an extension of a plaque in the carotid bulb or as an isolated involvement of the PHA, as it shares similar flow dynamics to those of the carotid bifurcation.^10^ Debris from atherosclerotic plaque in internal carotid artery with PHA can present with an atypical ischemic pattern involving the anterior as well as the posterior circulation.^9^ Abnormal exposure of the basilar artery to the hemodynamic stress of the internal carotid artery due to PHA has been postulated as a mechanism of development of an aneurysm in the posterior circulation. In our case, there was also an aneurysm involving the right posterior cerebral artery. We therefore presume abnormal hemodynamic stress exerted on the posterior cerebral artery due to PHA was an etiologic factor in the dissecting aneurysm of the right posterior cerebral artery in our case.

Posterior cerebral artery aneurysms are uncommon, accounting for 1.2% of all intracranial aneurysms, and are often non-saccular and dissecting in nature.^11^^,^^12^ Dissecting aneurysms are diagnosed on the cerebral angiogram by the “string and pearl sign” indicating an aneurysm preceding or following a stenotic segment.^12^ Other less frequently encountered angiographic signs are presence of an intimal flap, a subintimal hematoma, or a double lumen. These aneurysms are different from other saccular aneurysms because they present in a younger subgroup of patients. The most common location of dissecting aneurysm of the posterior cerebral artery is its P2-P3 segment as this part traverses across the tentorium cerebri and stress on the wall of the vessel along tentorial edges could be a possible explanation for the predilection for this site.^13^ Dissecting posterior cerebral artery aneurysm can present either as subarachnoid hemorrhage, as mass effect symptoms such as hemianopia, hemianesthesia, and hemiparesis, or as ischemic symptoms in the posterior cerebral artery territory.^14^ Dissecting aneurysms can grow in size and the risk of rebleed is significant and warrants emergent treatment.^12^^,^^14^ Surgical treatment is associated with a risk of neurological complications because of the deep location, proximity to the cranial nerves and the upper brainstem structures, and the perforators at this anatomical location.^12^ Endovascular treatment is a favorable treatment option as it does not involve manipulation of adjacent vital structures. The primary goal of endovascular treatment is to occlude the aneurysm sac while preserving the patency of the parent artery. However, this is not always feasible in dissecting aneurysms and PVO is then the only endovascular option available.^12^ A balloon occlusion test can be performed before the PVO to ensure the safety of the procedure. In their review of 98 cases, Huang et al. found 16% symptomatic ischemic complications and no hemorrhagic complications.^15^

In our case, a young woman presented with occipital headache and was diagnosed with PHA with a dissecting aneurysm involving the P2-P3 segment of the right posterior cerebral artery, cerebral angiogram showed the string and pearl sign diagnostic of dissecting aneurysm, and we decided to perform PVO due to dissecting nature of aneurysm. We navigated through the PHA because both vertebral arteries were hypoplastic. Post-PVO, the patient did not develop any neurological deficits. Our case thus represents an example of PHA associated with aneurysm treated by navigating through the PHA. It also highlights the fact that PVO is a safer endovascular option in P2-P3 segment dissecting aneurysm.

## CONCLUSION

Endovascular treatment of dissecting posterior cerebral artery aneurysm associated with persistent hypoglossal artery is feasible. Navigation may be achieved via the persistent hypoglossal artery in case of bilateral hypoplasia of vertebral arteries. Parent vessel occlusion could be a safe and effective treatment option in these cases.
